# Heterostructure Composites of CoS Nanoparticles Decorated on Ti_3_C_2_T_x_ Nanosheets and Their Enhanced Electromagnetic Wave Absorption Performance

**DOI:** 10.3390/nano10091666

**Published:** 2020-08-26

**Authors:** Hui Liu, Ling Li, Guangzhen Cui, Xinxin Wang, Zhi Zhang, Xuliang Lv

**Affiliations:** 1 Graduate School, The Army Engineering University of PLA, Nanjing 210007, China; liuhh1005@163.com (H.L.); 18260093995@163.com (X.W.); zhangnjjn@163.com (Z.Z.); 2 Engineering College of Field Engineering, The Army Engineering University of PLA, Nanjing 210007, China; xllu1957@126.com

**Keywords:** MXene Ti_3_C_2_T_x_, CoS nanoparticle, dielectric loss, microwave absorption

## Abstract

As a typical two-dimensional material, MXene possesses excellent conductivity and tunable interlayer space, which makes it have an impressive development potential in the field of electromagnetic (EM) waves absorbing materials. In this work, we fabricated a sandwich structure CoS@Ti_3_C_2_T_x_ composite using a simple solvothermal process. The CoS nanoparticles are anchored on the Ti_3_C_2_T_x_ MXene sheets, forming a heterolayered structure. The results demonstrate that the CoS@Ti_3_C_2_T_x_ composites with the sandwich-like architecture showed excellent EM absorbing performance due to the synergistic effects of the conductivity loss, interface polarization, and dipole polarization. When the doping ratio was 40 wt %, the maximum reflection loss value of CoS@Ti_3_C_2_T_x_ was up to –59.2 dB at 14.6 GHz, and the corresponding effective absorption bandwidth (below –10 dB) reached 5.0 GHz when the thickness was only 2.0 mm. This work endows a new candidate for the design of MXene-based absorption materials with optimal performance.

## 1. Introduction

Electromagnetic (EM) pollution has emerged from the explosive development of military equipment, and communication technology has produced serious damage for human beings [1,2]. Thus, microwave absorption materials have attracted increasing attention, which effectively converts the EM energies into thermal energies [3,4]. Strong EM wave attenuation intensity and wide effective bandwidth are the pursuits for the preparation of superior absorbing materials [5]. Meanwhile, light weight and high efficiency are also two key factors affecting their wide application in daily life [6,7]. To date, a great number of absorbing materials have been reported such as carbon materials (nanoporous carbons, graphene, CNTs, carbon fibers), conductive polymers (PPy, PANI), and semiconductor transition-metal sulfides (CuS, MoS_2_), etc. Mohd Najim et al. used nickel–phosphorus coating on the tetrapod-shaped ZnO by the electroless coating process. The Ni–P coating increased the magnetic loss of the material, and the maximum reflection loss (RL) of the Ni–P coated T–ZnO reached –36.41 dB with an effective absorption bandwidth of 10.0 GHz [8]. Wang et al. designed a CoFe_2_O_4_/N-RGO aerogel wherein CoFe_2_O_4_ was embedded in the N-doped reduced graphene oxide (RGO) aerogels and the strongest RL was –60.4 dB at 14.4 GHz [9].

Notably, MXene, a typical 2D structure composed of transition-metal carbides, nitrides, and/or carbonitrides, has gained interest in the field of EM wave absorbing [10]. Due to their special laminated structure, high conductivity, and huge specific surface area, MXene exhibits huge potential application in lithium-ion batteries [11], supercapacitors [12], EM interference shielding [13], and EM wave absorbers [14]. As we all know, Ti_3_C_2_T_x_, where T_x_ represents the surface terminations (such as –OH, =O, –F) after etching by hydrofluoric acid, is the first MXene material discovered by Yury Gogotsi [15,16,17]. These surface functional groups will induce extra dipoles to generate dipole polarization, which may enhance the dielectric loss and optimize impedance matching [18]. Nevertheless, the impedance matching of sole MXene is poor due to the high conductivity, which cannot meet the absorption requirements of strong absorption, broad bandwidth, light-weight, and thin thickness. For instance, Zhang et al. fabricated Ti_3_C_2_T_x_ MXene materials; when the filler loading was up to 50 wt %, the maximum RL of Ti_3_C_2_T_x_ MXene was –29.6 dB with a thickness of 1.8 mm and the effective absorption bandwidth was less than 3 GHz [19]. Previous studies have confirmed the principle that the combination of MXene and other materials can effectively construct the heterogeneous, which is beneficial to attenuate the EM energies such as Ti_3_C_2_T_x_@RGO [20], Ti_3_C_2_T_x_@Ni [21], Ti_3_C_2_T_x_/CNTs [22], and Ti_3_C_2_T_x_@poly (vinyl alcohol) [23]. Among them, the composite of Ti_3_C_2_T_x_ and nanomaterials has been found to further improve the absorption performance. Liu et al. prepared TiO_2_/Ti_3_C_2_T_x_/Fe_3_O_4_ composites by the hydrothermal process [24]. The ultrasmall sized Fe_3_O_4_ introduces an abundant surface area, which increases the multiple scattering and reflection between the Ti_3_C_2_T_x_ interlayers. The maximum RL was –57.3 dB (at 10.1 GHz) and the corresponding thickness was 1.9 mm. However, the effective absorption bandwidth of the TiO_2_/Ti_3_C_2_T_x_/Fe_3_O_4_ composites was only 2.1 GHz (6.6–8.7 GHz) while the doping ratio of powder in paraffin was up to 70 wt %, indicating that further optimization is needed in the terms of the absorption bandwidth and weight. Cui et al. reported that the two-dimensional (2D) Ti_3_C_2_T_x_ modified with CuS nanoparticles exhibited a minimum RL value of –43.5 dB at 2.0 mm, in which the effective absorption bandwidth was up to 5.2 GHz [25]. It was confirmed that MXene can be combined with transition metal sulfide for application in absorbing materials.

Cobalt sulfide (CoS), as a semiconductor metal sulfide, exhibits superior theoretical capacity and good electrical properties in the fields of supercapacitors and lithium batteries [26,27,28,29]. In recent years, CoS hybrid absorption composites have attracted much attention. Huang et al. prepared a heterostructure MWCNTs/CoS material, which consisted of numerous CoS nanoplates anchored with MWCNTs, and obtained good EM wave absorption performance [30]. Our groups fabricated cole-shell structure CoS@ppy composites, which showed an optimal RL of –41.8 dB at 6.96 GHz with a filler loading of 20 wt % [31]. In addition, Zhang et al. fabricated a CoS/MXene composite that showed outstanding electrochemical performance, and the MXene, acting like a circuit plate, provided a conductive network for CoS [32]. It has been considered to combine high conductivity Ti_3_C_2_T_x_ MXene with CoS nanoparticles to synthesize absorption materials. To our knowledge, no such reports have been reported. The laminated structure of MXene can increase the EM wave propagation path, which is beneficial to enhance the attenuation of EM waves through scattering. As a result of the negative charges reaction, Co^2+^ ions can be absorbed on the surface of MXene. Moreover, the defects will act as polarization centers and induce polarization relaxation under alternating electromagnetic fields. The introduction of nanoscale particles into the material can be beneficial to increase the polarization loss and enhance the absorption of EM waves. Considering the excellent multiple structure of Ti_3_C_2_T_x_ nanosheets and the greatly increased interfaces induced by the CoS composites, it is significant to explore the CoS@Ti_3_C_2_T_x_ MXene hybrid for EM wave absorption.

Herein, in this study, sandwich-like CoS@Ti_3_C_2_T_x_ MXene composites were successfully prepared via a solvothermal method and first used as a high-efficiency EM wave absorber. The morphology, crystalline structure, and EM absorption properties of CoS@Ti_3_C_2_T_x_ MXene materials were investigated. Moreover, the attenuation mechanisms of EM waves were also illustrated such as dipole polarization, interfacial polarization, dielectric loss, and impedance matching. The results indicate that the CoS/Ti_3_C_2_T_x_ MXene composites is a potential candidate for the EM wave absorbing composite, which possesses strong absorption and broad bandwidth.

## 2. Materials and Methods

### 2.1. Materials

Ti_3_AlC_2_ powders (purity > 99%, 200 mesh) were obtained from Changchun 11 Technology Co. Ltd. (Changchun, China). Lithium fluoride (LiF) (purity > 99%) and hydrochloric acid (HCl) (36–38%) were obtained from Macklin Technology Ltd. (Shanghai, China). Ethylene glycol (EG, AR), cobalt chloride hexahydrate (CoCl_2_·6H_2_O, AR), and thiourea (CN_2_H_4_S, AR) were provided from Aladdin Technology Co. Ltd. (Shanghai, China).

### 2.2. Synthesis of Ti_3_C_2_T_x_ MXene

Ti_3_C_2_T_x_ MXene was synthesized based on the previous work [33]. First, 1 g LiF was completely dissolved in 20 mL of 9 M HCl solution with stirring for 5 min at room temperature. Then, 1 g Ti_3_AlC_2_ powder was slowly added into the above solution and kept stirring at 45 °C for 24 h. Subsequently, the homogeneous mixture was washed three times using deionized water by centrifuge (3500 rpm) until the pH reached 6. Finally, the obtained black samples were dried in the desiccator under vacuum at 50 °C.

### 2.3. Synthesis of CoS@Ti_3_C_2_T_x_ Hybrids

**Figure 1:**
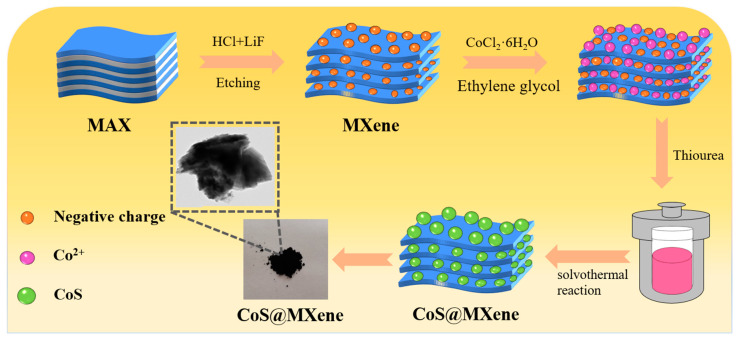
Schematic diagram of the synthesis of CoS@Ti_3_C_2_T_x_ composites.

The synthesis of CoS@Ti_3_C_2_T_x_ hybrids was carried out in a solvothermal reaction. In detail, 200 mg Ti_3_C_2_T_x_ MXene was dissolved in 20 mL EG by ultrasound for 1 h. Then, 2.5 mmol CoCl_2_·6H_2_O was dispersed in 30 mL EG and mixed with the former solution, stirring for 30 min. Subsequently, the 20 mL EG solution dissolved in 6.25 mmol thiourea was slowly added into the above solution with magnetic stirring for 30 min. The homogeneous suspension was moved into the 100 mL Teflon-lined autoclave (Shanghai Yuezhongyq Co.Ltd, Shanghai, China) and reacted at 180 °C for 12 h. Meanwhile, CoS nanoparticles were prepared for comparison. The schematic illustration of the synthesis of CoS@Ti_3_C_2_T_x_ hybrids is shown in Figure 1.

### 2.4. Characterization

The microscopic morphology of Ti_3_C_2_T_x_ MXene, CoS nanoparticles, and CoS@Ti_3_C_2_T_x_ composite were detected by scanning electron microscopy (SEM, JSM-7800F, JEOL, Tokyo, Japan). The microstructure and elemental mapping were characterized by an energy dispersive x-ray spectroscopy (EDS, XFlash 5030T, BRUKER, Leipzig, Germany) and a transmission electron microscope (TEM, JEM-2100F, JEOL, Tokyo, Japan) with a scanning transmission electron microscope (STEM) resolution of 0.20 nm. X-ray diffraction (XRD, D8A Advance, BRUKER, Leipzig, Germany) was used to analyze the crystallite structure of the composites. The surface of the CoS@Ti_3_C_2_T_x_ composite was performed by x-ray photoelectron spectroscopy (XPS, ESCALAB 250 xi, Shanghai, China). A vector network analyzer (VNA, N5242A PNA-X, Agilent, Palo Alto, CA, USA) was used to collect the basic EM parameters in the frequency range of 2.0–18.0 GHz at room temperature. The composite was mixed with paraffin in different filler ratios (35 wt %, 40 wt %, 45 wt %) and then pressed into a coaxial cylinder ($\Phi_{in}$ = 3.04 mm, $\Phi_{out}$ = 7.0 mm, d = 3.5 mm) under a pressure of 5 MPa.

## 3. Results and Discussion

### 3.1. Characterization of Samples

**Figure 2:**
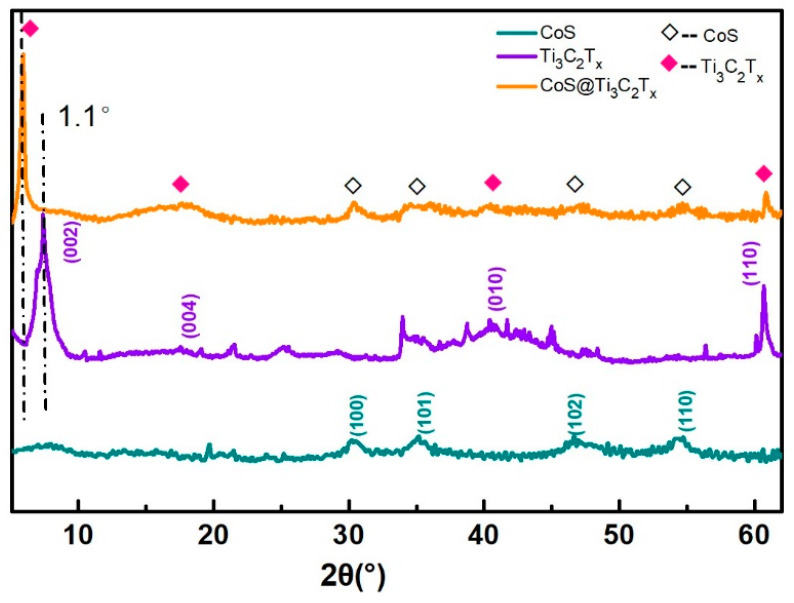
XRD patterns of CoS, MXene, and CoS@Ti_3_C_2_T_x_.

The x-ray diffraction (XRD) patterns of CoS, Ti_3_C_2_T_x_, and CoS@Ti_3_C_2_T_x_ are shown in Figure 2. The typical peaks at 7.2°, 17.6°, 42.0°, and 60.8° corresponded to the (002), (004), (010), and (110) crystal planes of Ti_3_C_2_T_x_, respectively [34]. Obviously, the (002) peak of CoS@Ti_3_C_2_T_x_ shifted to 6.1° after the solvothermal reaction. According to the Bragg equation, the layer space of Ti_3_C_2_T_x_ increased from 12.2 Å to 14.4 Å, indicating the cobalt sulfide nanoparticles had anchored on the Ti_3_C_2_T_x_ MXene layers to form a multilayered structure. In addition, the peaks at 30.2°, 35.14°, 46.66°, and 54.72° corresponded to the (100), (101), (102,) and (110) planes of hexagonal CoS (JCPDS No.75-0605), which indicates the successful synthesis of CoS@Ti_3_C_2_T_x_ composites.

**Figure 3:**
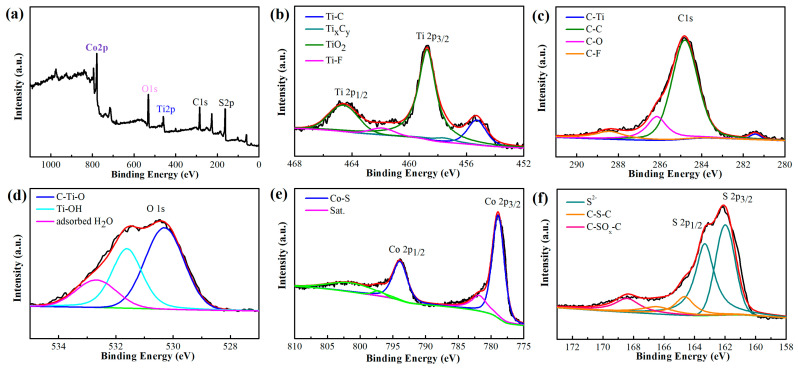
X-ray photoelectron spectroscopy (XPS) survey spectra of CoS@Ti_3_C_2_T_x_: (**a**) survey spectrum, (**b**) Ti 2p, (**c**) C 1s, (**d**) O 1s, (**e**) Co 2p, and (**f**) S 2p.

To further research the surface chemical elements of CoS@Ti_3_C_2_T_x_ composites, the XPS spectra are shown in Figure 3. As shown in Figure 3a, the total spectrum of the CoS/Ti_3_C_2_T_x_ composite demonstrates the existence of Co, Ti, O, S, and C elements. Figure 3b illustrates the Ti 2p XPS spectrum of the sample and the peaks corresponding to Ti–C (454.8 eV), Ti_x_C_y_ (457.7 eV), Ti 2p_3/2_ (458.3 eV), Ti–F (461.4 eV), and Ti 2p_1/2_ (464.1 eV) [35]. The C 1s spectra exhibited in Figure 3c contains four fitted peaks including Ti–C (281.4 eV), C–C (284.7 eV), C–O (286.3 eV), and C–F (288.7 eV) [36]. The O 1s peaks at 530.3 eV and 531.8 eV can be indexed to C–Ti–O and Ti–OH in Figure 3d [37]. Moreover, the peak at 532.8 eV confirms that there are a small number of water molecules in the Ti_3_C_2_T_x_ MXene layers [38]. In Figure 3e, the peak at 793.8 eV is indexed to Co 2p_1/2_ and the peak located at 778.8 eV belongs to Co 2p_3/2_ [39]. In addition, the presence of C–S–C, C–SO_x_–C, S 2p_1/2_, and S 2p_3/2_ can be observed in Figure 3f. Thus, the XPS analysis indicates that the heterogeneous structural CoS@Ti_3_C_2_T_x_ composites were prepared, which also corresponded to the XRD analysis.

**Figure 4:**
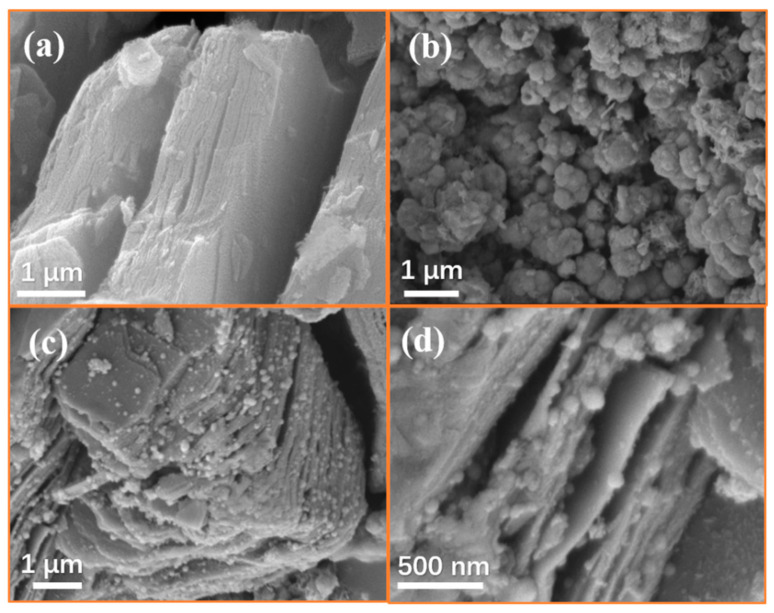
Scanning electron microscopy (SEM) of Ti_3_C_2_T_x_ (**a**), CoS nanoparticles (**b**), CoS@Ti_3_C_2_T_x_ composite (**c**,**d**).

**Figure 5:**
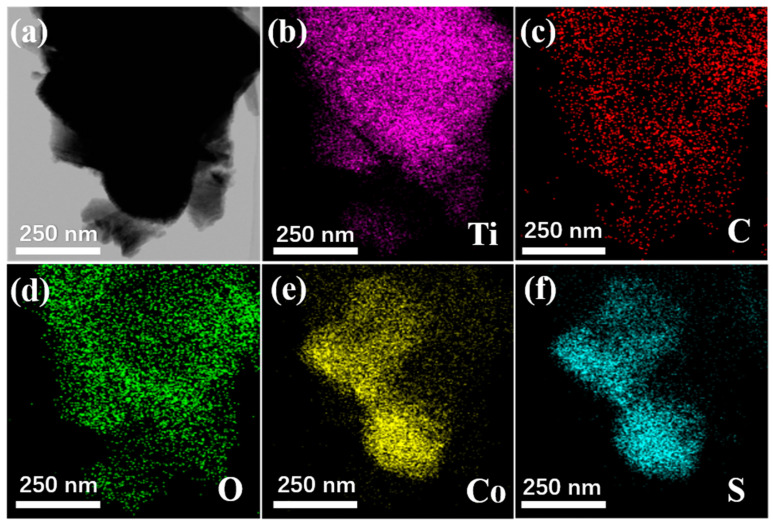
TEM of CoS@Ti_3_C_2_T_x_ (**a**), and its corresponding elemental mapping images of Ti (**b**), C (**c**), O (**d**), Co (**e**), and S (**f**).

**Figure 6:**
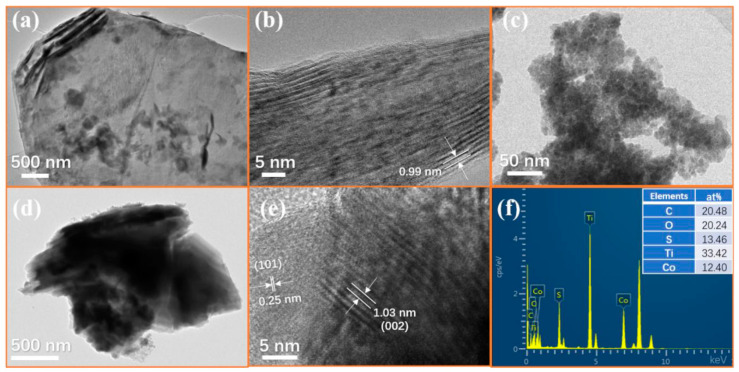
TEM (**a**), HRTEM (**b**) images of Ti_3_C_2_T_x_, TEM image of CoS nanoparticles (**c**), TEM (**d**), HRTEM (**e**) and corresponding EDS (**f**) images of CoS@Ti_3_C_2_T_x_.

The morphology of Ti_3_C_2_T_x_ MXene is given in Figure 4a. It can be observed that Ti_3_C_2_T_x_ MXene showed a similar accordion-like structure after etching. A large number of agglomerated CoS nanoparticles can be observed in Figure 4b. As shown in Figure 4c,d, CoS nanoparticles are anchored on the surface and inside the Ti_3_C_2_T_x_ MXene, forming a sandwich structure. It is worth noting that the interlayer spacing of the composites is significantly larger than pure MXene. From Figure 4b, it can be found that there was obvious agglomeration of pure CoS nanoparticles. As shown in Figure 4c, when CoS nanoparticles were combined with the two-dimensional material Ti_3_C_2_T_x_ MXene, Co^2+^ ions can be absorbed and dispersed by the functional groups on the surface of MXene, which effectively solved the agglomeration of CoS nanoparticles. These CoS nanoparticles will connect with Ti_3_C_2_T_x_ nanosheets to form a conductive network, which may be beneficial to increase the dielectric loss of the material. Figure 5 shows the TEM of the CoS@Ti_3_C_2_T_x_ composites, from which a typical laminated structure of the Ti_3_C_2_T_x_ MXene can be observed. It can be seen from the elemental mapping images that the Ti, C, and O elements were uniformly distributed in the diagram. Due to the oxidation on the surface of the Ti_3_C_2_T_x_ MXene, the oxygen element was detected. The distribution of Co and S further confirms the successful composition of the CoS nanoparticles and Ti_3_C_2_T_x_ MXene. Moreover, the TEM image of the Ti_3_C_2_T_x_ MXene in Figure 6a shows that Ti_3_C_2_T_x_ MXene presents an ultrathin transparent laminated structure and the interlayer space of the Ti_3_C_2_T_x_ MXene was approximately 0.99 nm, as shown in the high resolution transmission electron microscope (HRTEM) image in Figure 6b. The average diameter of CoS nanoparticles was about 15–17 nm and it can be observed that these nanoparticles were embedded in the interlayer or surface of the Ti_3_C_2_T_x_ MXene nanosheets. As described in Figure 6e, the interlayer spacing of 0.25 nm and 1.03 nm corresponded to the (101) facets of the CoS nanoparticles and the (002) planes of the laminated Ti_3_C_2_T_x_ MXene, respectively. In order to further demonstrate the existence of CoS nanoparticles on the surface of Ti_3_C_2_T_x_ MXene, the corresponding EDS image is shown in Figure 6f. It can be seen that the five elements of Ti, Co, S, O, C were detected, and the atomic ratio of Co and S was around 1:1, which corresponds to the stoichiometry of CoS. Moreover, no other elements were found, which further confirms the successful preparation of CoS@Ti_3_C_2_T_x_ composites.

### 3.2. Electromagnetic Parameters and Absorption Property

**Figure 7:**
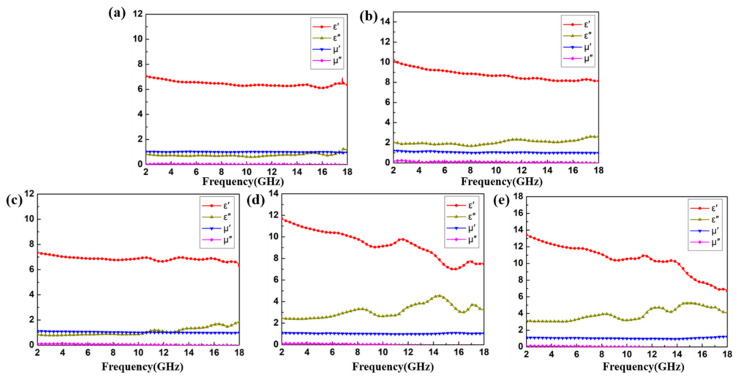
Real and imaginary parts of the complex permittivity and permeability of Ti_3_C_2_T_x_ (35 wt %) (**a**), CoS (35 wt %) (**b**), CoS@Ti_3_C_2_T_x_ (35 wt %) (**c**), CoS@Ti_3_C_2_T_x_ (40 wt %) (**d**), and CoS@Ti_3_C_2_T_x_ (45 wt %) (**e**).

To evaluate the EM wave absorption characteristics, the relative complex permittivity (ε_r_ = ε′–jε″) and relative complex permeability (μ_r_ = μ′–jμ″) of the Ti_3_C_2_T_x_, CoS, and CoS@Ti_3_C_2_T_x_ MXene composites with different filler loading were measured using a vector network analyzer ground on the coaxial-line method in the frequency range of 2–18 GHz. The samples were mixed with paraffin and pressed into a ring model ($\Phi_{in}$ = 3.04 mm, $\Phi_{out}$ = 7.0 mm), which were then placed in a coaxial clamp. After multiple reflection and transmission between the air interface of the transmission line and the sample, the EM wave energy would attenuate and the phase would shift. Then, the scattering parameter S is measured by a vector network analyzer and the EM parameters can be calculated according to the standard Nicolson–Ross–Weir theory [40,41]. In general, the real part of the relative complex permittivity (ε′) represents the polarization capability of the composite in the electric field, the real part of the relative complex permeability (μ′) shows magnetization capability under the influence of a magnetic field. The imaginary part of the relative complex permittivity (ε″) and the relative complex permeability (μ″) represent dielectric loss and magnetic loss capacity, respectively [42]. As shown in Figure 7a, the average ε′ and ε″ value of Ti_3_C_2_T_x_ (35 wt %) were maintained at 6.9 and 0.8, respectively. In comparison, the average ε′ and ε″ values of CoS (35 wt %) reached 10.0 and 2.0, respectively, as shown in Figure 7b. As the filler loading in the paraffin matrix increased from 35 wt % to 45 wt %, the ε′ and ε″ values of CoS@Ti_3_C_2_T_x_ added up to 13.5 and 5.1, respectively, which may be illustrated by the effective medium theory [43]. The dipole polarization, interfacial polarization, and electrical conductivity may be enhanced by the increase in CoS@Ti_3_C_2_T_x_ weight [19]. As demonstrated in Figure 7d,e, it is worth noting that the ε′ and ε″ curves fluctuated significantly within the 8–18 GHz, and it may be related to the relaxation polarization and interfacial polarization of dielectric materials at high frequencies. In particular, the ε′ and ε″ achieved the highest values, indicating that CoS@Ti_3_C_2_T_x_ (45 wt %) possibly has a favorable dielectric dissipation capability to EM waves, as shown in Figure 7e. Moreover, as shown in Figure 7, due to the absence of magnetism in these composites, the μ′ and μ″ values reached 1.0 and 0, respectively. The above analysis showed that dielectric loss is the major mechanism of EM wave absorption in the CoS@Ti_3_C_2_T_x_ composites, while the magnetic loss can be ignored.

**Figure 8:**
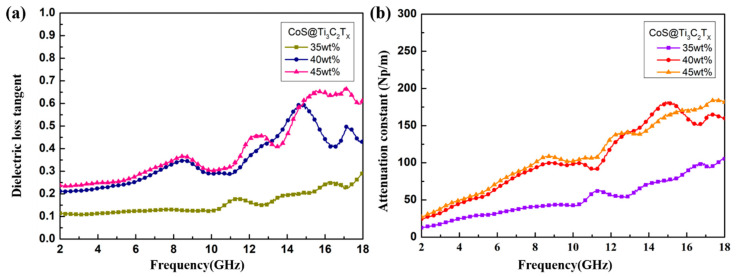
Dielectric loss tangent (**a**) and attenuation constant (**b**) of CoS@Ti_3_C_2_T_x_ composites with different filler loadings.

Dielectric loss is related to two important factors: polarization relaxation and conductivity loss, and the dielectric loss tangents ($tan\;\delta_{\varepsilon }=\varepsilon^{\prime \prime }/\varepsilon^{\prime }$) of CoS@Ti_3_C_2_T_x_ composites with different filler loadings are calculated in Figure 8a. With the increase in frequency, the $\tan\delta_{\varepsilon }$ curves showed an upward trend and some vibration peaks corresponded well to the permittivity curves. The average values of curves at 40 wt % and 45 wt % increased from 0.25 to 0.35 at 2–10 GHz, respectively. However, as the frequency continued to increase, the value of the 45 wt % curve achieved 0.65, indicating better dielectric loss capability. A similar phenomenon can be seen in the attenuation constant (α) curves in Figure 8b, which can be calculated as follows [44]:

(1) $$\alpha =\frac{\sqrt{2}\pi f}{c}\times \sqrt{\left(\mu^{\prime \prime }\varepsilon^{\prime \prime }-\mu^{\prime }\varepsilon^{\prime }\right)+\sqrt{{\left(\mu^{\prime \prime }\varepsilon^{\prime \prime }-\mu^{\prime }\varepsilon^{\prime }\right)}^{2}+{\left(\mu^{\prime }\varepsilon^{\prime \prime }+\mu^{\prime \prime }\varepsilon^{\prime }\right)}^{2}}}$$

where f is frequency and c represents the velocity of light. The larger *α* value means a stronger EM wave dissipation ability. As shown in Figure 8b, CoS@Ti_3_C_2_T_x_ (35 wt %) had a significantly lower absorption of EM waves than 40 wt % and 45 wt %. To further investigate the polarization relaxation phenomenon of CoS@Ti_3_C_2_T_x_ composites, the Cole–Cole semicircle model is necessary. The Debye equation is as follows [45]:

(2) $$\varepsilon_{r}=\varepsilon^{\prime }+i\varepsilon^{\prime \prime }=\varepsilon_{\infty }+\frac{\varepsilon_{s}-\varepsilon_{\infty }}{1+i\omega \tau }$$

According to Equation (2), the *ε*′ and *ε*″ can be expressed as:

(3) $$\varepsilon^{\prime }=\varepsilon_{\infty }+\frac{\varepsilon_{s}-\varepsilon_{\infty }}{1+\omega^{2}\tau^{2}}$$

(4) $$\varepsilon^{\prime \prime }=\frac{\omega \tau \left(\varepsilon_{s}-\varepsilon_{\infty }\right)}{1+\omega^{2}\tau^{2}}$$

where $\varepsilon_{s}$ stands for the static permittivity; $\varepsilon_{\infty }$ stands for the relative dielectric constant; τ is the polarization relaxation time; and ω stands for the electric field oscillation frequency. According to Equations (3) and (4), the relationship between *ε*′ and *ε*″ may be described by [46]:

(5) $${\left(\varepsilon^{\prime }-\frac{\varepsilon_{s}+\varepsilon_{\infty }}{2}\right)}^{2}+{\left(\varepsilon^{\prime \prime }\right)}^{2}={\left(\frac{\varepsilon_{s}-\varepsilon_{\infty }}{2}\right)}^{2}$$

**Figure 9:**
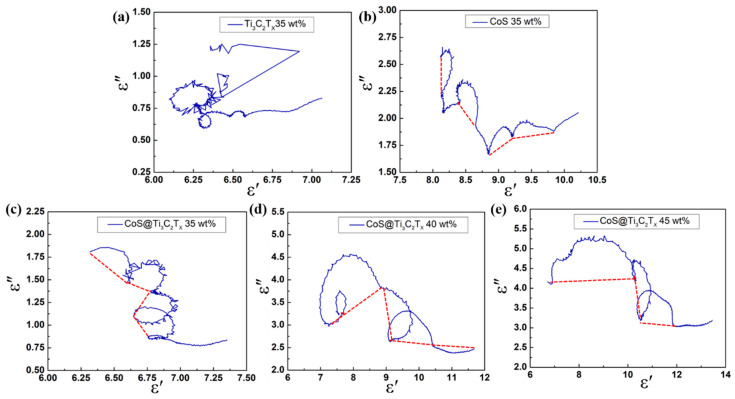
Typical Cole–Cole plots of Ti_3_C_2_T_x_ (35 wt %) (**a**), CoS (35 wt %) (**b**), CoS@Ti_3_C_2_T_x_ (35 wt %) (**c**), CoS@Ti_3_C_2_T_x_ (40 wt %) (**d**), and CoS@Ti_3_C_2_T_x_ (45 wt %) (**e**).

The plot of *ε*′ versus *ε*″ can be expressed as a Cole–Cole semicircle and each Cole–Cole semicircle corresponds to a polarization relaxation process [47]. In Figure 9a, because of the effect of the multi-relaxation dielectric properties, the Cole–Cole semicircle of Ti_3_C_2_T_x_ (35 wt %) showed a complex interlacing shape. In Figure 9b, there are four small distorted semicircles in CoS (35 wt %). The reason of semicircle distortion is that the Debye equation is an ideal model built under special conditions, and there are lattice distortion and point defects in the material [48]. In Figure 9c–e, three or four distinct semicircles can be observed in the CoS@Ti_3_C_2_T_x_ composites, which may result from the synergistic effects of dipole polarization and interfacial polarization [49]. More dipoles can be induced by the localized defects, oxygen functional groups, and multilayered structure of Ti_3_C_2_T_x_ [17,50,51]. Furthermore, there are many CoS nanoparticles embedded on the surface of the Ti_3_C_2_T_x_. According to the nanometer size effect, the number of dangling bonds, dipoles, and defects in the CoS@Ti_3_C_2_T_x_ composites would increase significantly, which may obviously enhance the electronic polarization and dipole polarization [52,53]. In addition, based on the Maxwell–Wanger–Sillars (MWS) effect, the special multicomponent heterostructure might generate more interfacial polarization process and thus enhance the absorption capability of EM waves [54].

**Figure 10:**
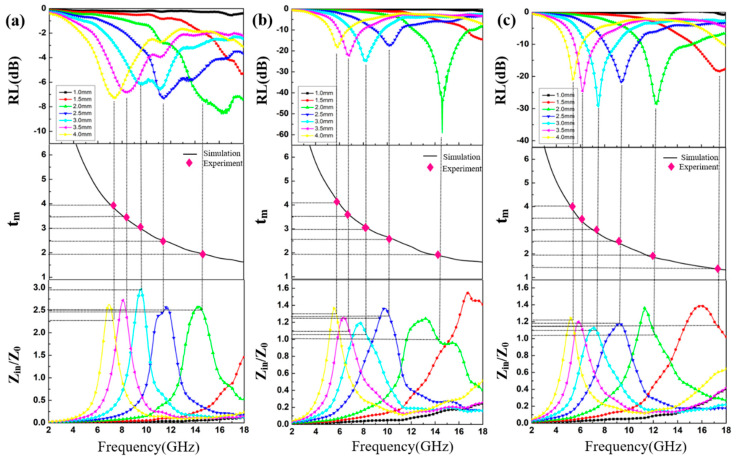
The RL curves, the matching thickness (*t_m_*) under λ/4 conditions and impedance matching of CoS@Ti_3_C_2_T_x_ with different filler loadings: 35 wt % (**a**), 40 wt % (**b**), and 45 wt % (**c**).

In order to further research the EM wave absorption characteristics of the CoS@Ti_3_C_2_T_x_ composites, the reflection loss (RL) values versus frequency of the materials with different filler loadings at specific thickness are shown in Figure 10. As described by transmission line theory, the RL values can be calculated by [55]:

(6) $$Z_{in}=Z_{0}\sqrt{\frac{\mu_{r}}{\varepsilon_{r}}}\tan h\left[j\frac{2\pi fd}{c}\sqrt{\mu_{r}\varepsilon_{r}}\right]$$

(7) $$\mathrm{RL}=20\log\left|\frac{Z_{in}-Z_{0}}{Z_{in}+Z_{0}}\right|$$

where *d* denotes the thickness of the absorbers; and *Z_in_* and *Z*_0_ stand for the normalized input characteristic impendence and the impendence of air, respectively. Figure 10a illustrates that the maximum RL values of CoS@Ti_3_C_2_T_x_ (35 wt %) at different thickness were above −10 dB, which means that the CoS@Ti_3_C_2_T_x_ (35 wt %) cannot absorb EM waves effectively. Comparatively, the maximum RL value of CoS@Ti_3_C_2_T_x_ (40 wt %) was −59.2 dB at 14.6 GHz and the corresponding optimal thickness was 2.0 mm, and the effective absorbing bandwidth was 5.0 GHz (12.24–17.24 GHz), as given in Figure 10b. When the filler ratio was 45 wt %, the maximum RL value of −28.83 dB at 12.32 GHz was found at a thickness of 2.0 mm, while the absorbing bandwidth below −10 dB was 4.16 GHz (11.04–15.2 GHz). After analysis, it can be found that the CoS@Ti_3_C_2_T_x_ (40 wt %) composite had superior EM absorption properties. Furthermore, it is worth noting that with the increase in the absorber thickness, the maximum RL locations shifted toward lower frequencies, which is consistent with the quarter-wavelength cancellation. The simulation curve of the absorption thickness (t_m_) can be calculated by the 1/4 wavelength cancellation equation ($t_{m}=n\lambda /4=nc/(4f_{m}\sqrt{\left|\mu_{r}\right|\left|\varepsilon_{r}\right|}$)) [23]. In Figure 10, the pink dots represent the matching thickness. It is interesting to observe that the pink dots were accurately distributed on the quarter-wavelength simulation curve, suggesting that the absorbing mechanism of the composite conforms to the 1/4 wavelength theory. In addition, good impedance matching is also a necessary condition for the material to have excellent absorption capability. The normalized characteristic impedance ($Z=\left|Z_{in}/Z_{0}\right|$) versus frequency is shown in Figure 10. Combined with the RL curves, the corresponding Z of the CoS@Ti_3_C_2_T_x_ (40 wt %) was close to 1 with a thickness of 2.0 mm, indicating that the material has optimal impedance matching and good EM wave absorbing potential.

**Figure 11:**
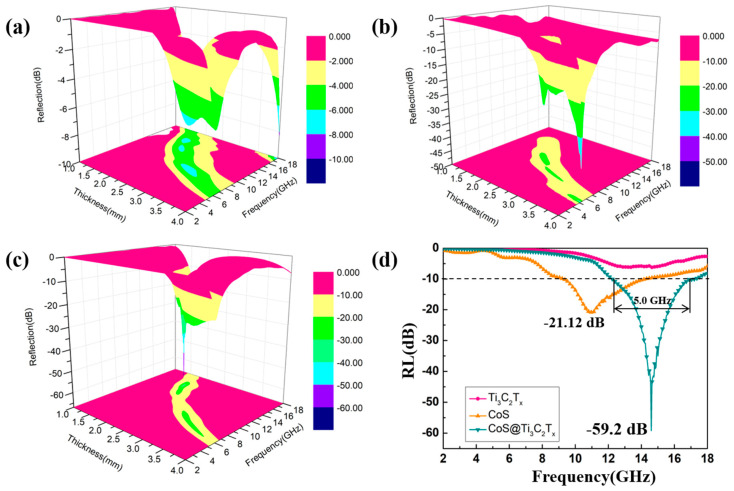
Three dimensional profiles of RL values of Ti_3_C_2_T_x_ (40 wt %) (**a**), CoS (40 wt %) (**b**), CoS@Ti_3_C_2_T_x_ (40 wt %) (**c**), and the RL curves at the thickness of 2.0 mm for them (**d**).

Figure 11 shows three dimensional profiles of RL values of Ti_3_C_2_T_x_, CoS and CoS@Ti_3_C_2_T_x_ at a 40 wt % doping ratio in the paraffin. From Figure 11a, the maximum RL value of Ti_3_C_2_T_x_ was −8.24dB and cannot be used as the EM wave absorber. In Figure 11b, the CoS nanoparticles were endowed with the maximum RL value of −39.44 dB and the responding thickness was 4.0 mm at 5.2 GHz. Although the absorption strength is acceptable, the absorption thickness cannot meet the requirement of an excellent EM wave absorbing material. In Figure 11c, the CoS@Ti_3_C_2_T_x_ composite exhibited the maximum RL value of −61.84 dB obtained at 14.3 GHz, and the absorbing thickness was only 1.84 mm. Moreover, when the thickness was 2.0 mm, the corresponding absorption bandwidth (RL < −10 dB) of the CoS@Ti_3_C_2_T_x_ composite reached 5.0 GHz (12.24–17.24 GHz), as shown in Figure 11d. Obviously, the CoS@Ti_3_C_2_T_x_ composite exhibited strong absorption intensity and broad effective bandwidth than that of the CoS and Ti_3_C_2_T_x_ composite due to their limited impedance matching.

**Figure 12:**
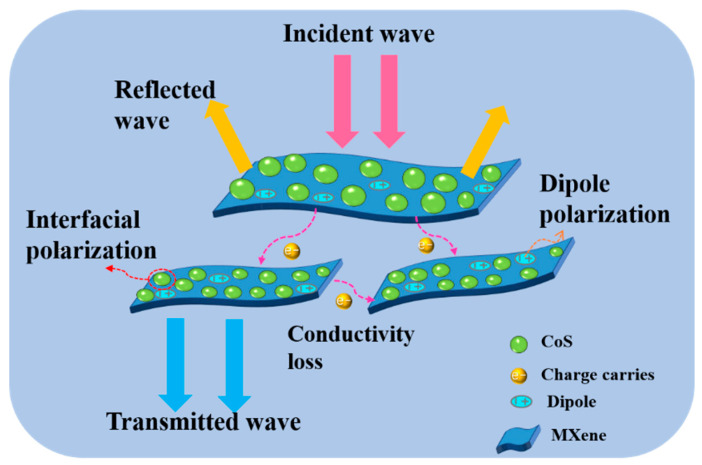
Scheme of EM wave absorbing mechanism of the CoS@Ti_3_C_2_T_x_ composites.

To further explain the attenuation process of EM waves in the CoS@Ti_3_C_2_T_x_ composite, a schematic diagram of the proposed absorption mechanism is given in Figure 12. First, due to good impedance matching, more incident EM waves could enter the material and be absorbed rather than reflected. Second, the unique sandwich structure of the CoS@Ti_3_C_2_T_x_ composite will expand the propagation path of EM waves inside the material, which may be conductive to the conversion of the EM waves into heat energy [56]. Meanwhile, based on the space-charge polarization effect, the interlayer space of the Ti_3_C_2_T_x_ MXene increased because of the existence of these nanoparticles, which perhaps benefits the enhancement of the absorption capacity [57,58]. Third, the introduction of CoS nanoparticles will significantly increase the conductive paths in the Ti_3_C_2_T_x_ MXene, carriers will migrate and hop between the Ti_3_C_2_T_x_ layers more actively. The formed field induced microcurrent may contribute to the conduction loss [59]. Moreover, abundant surface defects, dangling bonds, and functional groups (–F, –O, –OH) in Ti_3_C_2_T_x_ layers will form many polarized centers and generate a large number of dipoles, enhancing the dipolar polarization loss [60,61]. Finally, the interfacial polarization between CoS nanoparticles and Ti_3_C_2_T_x_ MXene sheets also favors the attenuation of EM waves. Thus, under the comprehensive influence of these factors, the CoS@Ti_3_C_2_T_x_ composite illustrates impressive absorption potential.

## 4. Conclusions

In this work, a CoS@Ti_3_C_2_T_x_ composite was successfully fabricated through a solvothermal reaction. After combining with Ti_3_C_2_T_x_ MXene, the impedance matching of the CoS@Ti_3_C_2_T_x_ composite had been significantly optimized. Enhanced dielectric loss, interfacial polarization, and unique sandwich structure also contributed to the EM wave absorption. As a result, the as prepared CoS@Ti_3_C_2_T_x_ composite showed excellent EM wave absorbing properties with the maximum RL value reaching −59.2 dB at an optimal thickness of only 2.0 mm. The effective absorbing bandwidth was up to 5.0 GHz (from 12.24 to 17.24 GHz). Therefore, our work offers an effective way to broaden the application fields for the development of other MXene-based absorbers.
